# Fast procedure for self-absorption correction for low γ energy radionuclide ^210^Pb determination in solid environmental samples

**DOI:** 10.1007/s10967-012-2404-8

**Published:** 2013-01-24

**Authors:** Magdalena Długosz-Lisiecka, Henryk Bem

**Affiliations:** Technical University of Lodz, Institute of Applied Radiation Chemistry, Zeromskiego Street 116, 90-924 Lodz, Poland

**Keywords:** Self-absorption correction, γ-Spectrometry system, Low γ energy radionuclides, Solid environmental samples

## Abstract

Low-energy X and γ radiations (for example of ^210^Pb: *E*_γ_ = 46.5 keV) are effectively self-absorbed even in thin environmental samples, including air filters with captured dust or contaminated soil, as well as in bottom sediment matrixes with limited quantities of the samples. In this paper, a simple method for the direct analysis of ^210^Pb (*T*_1/2_ = 22.3 years) by gamma-ray spectrometry in environmental samples with self-absorption correction is described. The method is based on the comparison of two γ peak activities coming from other natural radionuclides, usually present in environmental samples. We have analyzed the dependence of the self-absorption correction factor for the ^210^Pb activity on the activity ratios of 911 and 209 keV peaks and 609 and 295 keV peaks coming from nuclides of ^238^U or ^232^Th rows, present in typical environmental samples.

## Introduction

Long-lived natural radionuclide from uranium row ^210^Pb (*T*_½_ = 22.3 years) is widely used in radioecology [1], for example for aerosol residence time determinations [2] as well as the sedimentation rate or bottom sediments geochronology in different aquatic systems [3].

Instrumental γ spectrometry with HPGe detectors is usually applied for environmental radioactivity monitoring. The preferred method for the correcting of this effect is to use spiked [4] or natural matrix reference materials [5]. Commercially available radioactive standards allow us to establish the dependence of the detection efficiency versus the energy of γ-photons in the wide energy range from 40 to 2,000 keV, for the fixed geometry (for example: cylindrical or Marinelli beaker) [6] and known chemical composition of the sample. However, several very important primordial and anthropogenic radionuclides occurring in the environmental samples emit low-energy photons in the range up to 200 keV, particularly: ^210^Pb—46.5 keV, ^241^Am—59 keV, ^234^Th (^238^U)—63.3 and 92.6 keV, ^228^Th—84.8 keV, ^235^U—140, 163 and 186 keV and ^226^Ra—186 keV. For these radionuclides one should take into account the occurrence of the self-absorption of soft γ radiation in the measured samples, which strongly depends on the density, resultant atomic number—*Z* and geometry of the samples.

Therefore, instrumental gamma ray spectrometry may require additional corrections for self-absorption of gamma rays, as environmental samples often differ in densities and composition from each other, and the offered calibration standard reference materials may have slightly different chemical compositions. Generally, two basic approaches have been applied for solving the problem of self-attenuation in volume samples: experimental [6–12] and mathematical—using Monte Carlo simulations [13, 14]. Finally, a few computer programs have been developed for calculating the corrected detection efficiency for samples with a normalized shape with a known chemical composition (e.g. LabSOCS).

However, the sample geometry and efficiency calibration modeling by these methods is effective if one knows the exact chemical composition of the examined matrix. Practically, for example in the set of the bottom sediment samples or urban surface soil samples contaminated with heavy metals (Hg or Pb), even small changes in the concentration of these metals in basically the same matrixes can influence the resulting detection efficiency.

The aim of this study was develop an easy method for an additional detection efficiency correction factor for routine measurement of the soft γ emitters in the matrixes with slightly different heavy metal concentrations. The proposed procedure is based on the dependence of the activity ratio coming from the same radionuclide, or from the pair of radionuclides, in secular equilibrium usually present in typical environmental samples upon the self-absorption correction factor for the ^210^Pb activity. For these purposes we have chosen the pairs of 911 and 209 keV peaks or 609 and 295 keV peaks coming from nuclides of ^238^U or ^232^Th rows. A similar approach has been proposed by Haddad and Suman [15]. However, they have been using the pairs of radionuclides coming from different radionuclides, whose activity concentrations in the set of samples can vary. In our proposal, since the activity of the chosen pair of peaks results from the decay of the same natural radionuclide present in the sample, its ratio for a given geometry will depend only on the resulting atomic number of the matrix and geometry of the sample. In this way, chemical analysis of the matrixes is not necessary.

## Material and method

A coaxial HPGe detector GX3020 model with a beryllium window (maximal relative efficiency equal to 30 %, and FWHM about 2 keV for 1.33 MeV), housed in 10 cm Pb and 1 mm Cu shields (Canberra type) was used for low-background gamma spectrometry. Additionally, the preamplifier 2002-CSL type with a low noise FET input circuit ensured low background in the ^210^Pb detection region of 46.3. In this study, the Genie 2000 and LabSOCS (laboratory sourceless calibration system) software calibration tools were used. For evaluation of the mass attenuation, self-absorption coefficient and efficiency factors, the XCOM and ETNA software programs were also applied.

The IAEA Soil–Cu-2006-03 and Soil-327 standard reference materials (SRM) with a total mass sample of 50 g were chosen as starting matrixes for preparation of the set samples with increasing concentrations of heavy metals Hg and Pb, respectively. In these SRM’s there are natural radionuclides with certified activity concentrations from both uranium and thorium rows, emitting with sufficient intensity at least one pair of γ-rays each. For example: ^234^Th (63.4 and 92.6 keV), ^214^Pb (295 and 352 keV), ^214^Bi (609 and 1,120 keV) all from ^238^U row and ^228^Ac (209 and 911 keV) from ^232^Th row. The ^234^Th radionuclide with energies close to energy of 46.5 keV of ^210^Pb appeared to be the best to check the relationship between the ratio of its peak activity and the detection efficiency of the ^210^Pb radionuclide in different matrixes. However, the activities of both of these peaks are subjected to substantial interference from other natural radionuclides present in the environmental samples [16]. On the other hand, the energies of both ^214^Pb γ-rays are too close to each other and their ratio will change insignificantly. Additionally, the most abundant ^214^Bi-γ-lines are too high in comparison to 46.3 keV of ^210^Pb and the influence of the effective sample atomic number on the ratio of its activities in the environmental samples would not exemplify the changes in ^210^Pb detection efficiency. Fortunately, in the vast majority of environmental samples, a radioactive equilibrium between ^214^Pb and ^214^Bi is quickly established and 295 keV of ^214^Pb and 609 keV of ^214^Bi as well as the ^228^Ac 209 and 911 keV lines can be taken checking the proposed method. All the radionuclides used for ^210^Pb detection efficiency calibration are listed in the Table 1.

**Table 1:** Radionuclides used in analysis

Isotope	Energy (keV)	Decay efficiency (%)
Pb-210	46.5	4.05
Ac-228 (Th-228)	911.0	25.8
	209.0	4.1
Bi-214 (Ra-226)	609.4	45.0
Pb-214 (Ra-226)	295.2	18.7

A set of secondary standards were prepared by spiking the IAEA Soil–Cu-2006-03 and IAEA Soil 327 with various portions of Hg_2_Cl_2_ or Pb(NO_3_)_2_ solutions to get different Hg or Pb concentrations in this standard from 0 to 0.6 %. The samples after drying and weighting were inserted into a cylindrical polyethylene container with 80 mm of diameter and 7 mm height and sealed. The sample containers were placed directly on the detector before counting. In order to obtain an acceptably low statistical error, counting time *T* was 80,000 s.

## Results

In typical environmental samples self-absorption phenomena occurs almost always. The activity of nuclide *A* is derived from the formula (1):

1 $$ A = \frac{I}{{\varepsilon (E)\,\varepsilon (\gamma ){\kern 1pt} {\kern 1pt} \varepsilon (s)}}\quad $$

where *E* is the energy of photons emitted by the nuclide; *I* is the number of net count in a photopeak per second corresponding to energy *E*; ε(*E*) is the gamma-ray spectrometer detection efficiency for photons with energy *E* for a given geometry; *ε*(*γ*) is the gamma-ray emission probability for a measured radionuclide; *ε*(*s*) is self-absorption correction factor in the sample.Substituting

2 $$ \varepsilon_{\text{m }} = \varepsilon \left( E \right) \cdot \, \varepsilon \left( s \right) $$

where *ε*_m_ is the total detection efficiency of the gamma photons with energy of 46.5 keV, one gets the following expression

3 $$ A = \frac{I}{{\varepsilon_{\text{m}} \varepsilon (\gamma )}} $$

**Fig. 1:**
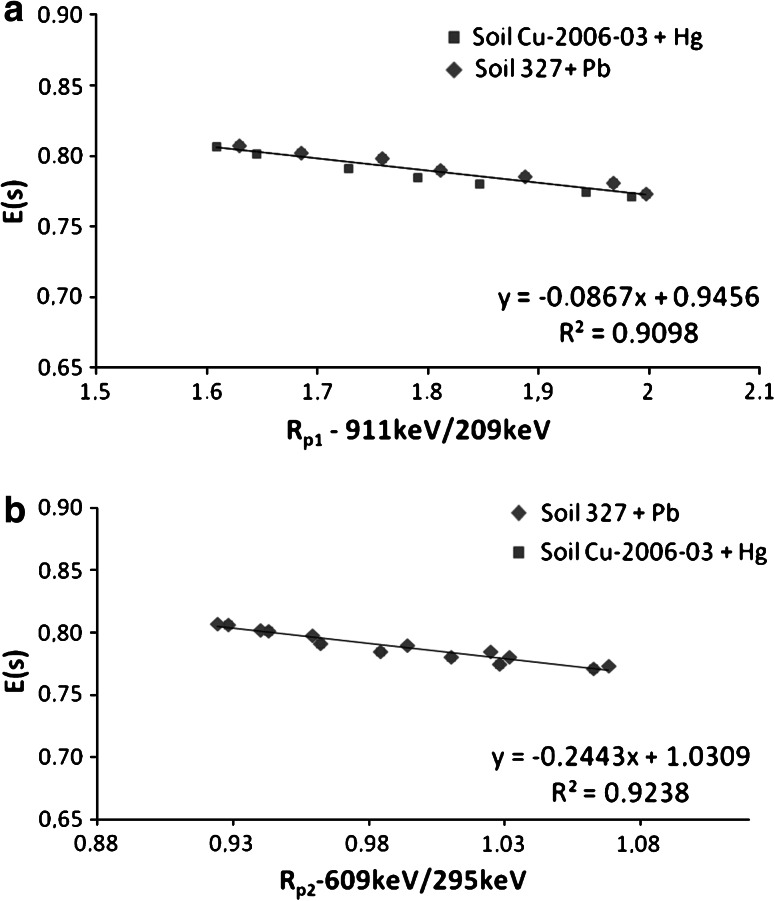
Dependence of the self-absorption coefficients on the activity ratio *R* of two pairs of the γ-lines **a** *R*_p1_ and **b** *R*_p2_ from natural radionuclides present in the environmental samples

For the samples with cylindrical geometry, placed directly on the surface of the germanium detector (Fig. 1) their measured activity for different thickness—*x* of the sample, can be calculated also after dissolving following equation.

4 $$ {\text{d}}I = A \cdot S \cdot \rho \cdot \varepsilon (E )\cdot \, \varepsilon (\gamma )\cdot {\text{d}}x{ - }\mu (E )\cdot I \cdot {\text{d}}x \, $$

where *A* is the specific activity of the measured radionuclide in the sample (Bq/g), *S* is surface of the sample (cm^2^), *ρ* is the density of the sample (g/cm^3^), *μ*(*E*) is the linear attenuation coefficient for photons with energy of *E* (MeV) (cm^−1^), *ε*_m_ is detection efficiency of the photons with energy *E* (keV) in the fixed geometry by the spectrometric system. This value is a product of the geometric efficiency and detector intrinsic efficiency for photons with quite a broad energy range. The value *ε*_m_ for normalized geometry of the samples can be calculated from the calibrated detector data. After integrating Eq. (2) one can get:

5 $$ I = A \cdot S \cdot \varepsilon (E) \cdot \varepsilon (\gamma ) \cdot \frac{1 - \exp ( - \mu x)}{\mu x} $$

or after substituting:

6 $$ S \cdot \rho \cdot x \, = \, m_{\text{S}} $$

where *x* is the thickness of the sample (cm), *m*_S_ is the mass of the sample (*g*)

7 $$ I = A \cdot m_{S} \cdot \varepsilon (E) \cdot \varepsilon (\gamma ) \cdot \frac{1 - \exp ( - \mu x)}{\mu x} $$

The last part of the Eq. (7) denotes the self-absorption factor *ε*(*s*).Therefore,

8 $$ \varepsilon (s) = \frac{1 - \exp ( - \mu x)}{\mu x} $$

and

9 $$ I \, = \, A \cdot m_{\text{S}} \cdot \varepsilon \left( E \right) \cdot \varepsilon \left( \gamma \right) \cdot \varepsilon \left( s \right) $$

or

10 $$ I \, = \, A \cdot m_{\text{S}} \cdot \varepsilon_{\text{m}} \cdot \varepsilon \left( \gamma \right) $$

During routine measurements of the set of the chemically identical samples in the fixed geometry, the values of *ε*(*E*)· *ε*(*γ*) ·*ε*(*s*) are constant and can be determined using the appropriate SRM’s or by available computer programs. However, very often such samples can differ slightly in terms of their different contamination with heavy metals. In this case the product of values *ε*(*E*)· *ε*(*s*) = *ε*_m_ e.g., the total detection efficiency of the soft γ photons can vary. For example, in the thick samples the self-absorption factor—*ε*(*s*) can change substantially, as the values of the linear attenuation coefficient—μ strongly depend on low energy X and γ-rays on the resultant atomic number—*Z*_R_ of the sample according to the formula:

11 $$ \mu \, = \, k \cdot \left( {Z_{\text{R}} } \right)^{n} $$

where *K* and *n* are constant depending on the value of the energy of the photonsand

12 $$ Z_{\text{R}} = \, \sum \, w_{\text{i}} \cdot Z_{\text{i}} $$

where *w*_i_, and *Z*_i_ denote weight shares and atomic numbers, respectively, for all elements in the samples.

Theoretically, knowing the exact chemical composition of the samples for *Z*_R_ calculation and on the basis of Eqs. (8) and (11), taking the necessary data from NIST tables [17] one can calculate the *ε*(*s*) values. However, the exact chemical analysis of the samples before radiometric measurements is troublesome, costly and time consuming. Therefore, we propose characterizing the chemical composition of each sample and, therefore, its self-absorption factors by simultaneous measurements of the activities of pairs of γ-lines coming from other natural radionuclides usually present in environmental samples, for example from ^222^Ac or ^214^Pb and ^214^Bi, together with ^210^Pb activity.

According to Eq. (7), the ratio of such pair activities *R*_P_ would be equal to:

13 $$ R_{\text{p}} = \frac{{A \cdot m_{\text{S}} \cdot \varepsilon (E_{ 1} )\cdot \varepsilon (\gamma_{ 1} )\cdot \frac{{ 1 {\text{ - exp( - }}\mu_{ 1} x )}}{{\mu_{ 1} x}}}}{{A \cdot m_{\text{S}} \cdot \varepsilon (E_{ 2} )\cdot \varepsilon (\gamma_{ 2} )\cdot \frac{{ 1 {\text{ - exp( - }}\mu_{ 2} x )}}{{\mu_{ 2} x}}}} $$

or after reduction:

14 $$ R_{\text{p}} = \frac{{\varepsilon (E_{ 1} )\cdot \varepsilon (\gamma_{ 1} )\cdot \frac{{ 1 {\text{ - exp( - }}\mu_{ 1} x )}}{{\mu_{ 1} }}}}{{\varepsilon (E_{ 2} )\cdot \varepsilon (\gamma_{ 2} )\cdot \frac{{ 1 {\text{ - exp( - }}\mu_{ 2} x )}}{{\mu_{ 2} }}}} $$

Therefore, the value of *R*_P_ for chosen γ lines and for fixed geometry is function only linear attenuation coefficients.

The latter depends on the chemical composition of the sample and *R*_P_ value can be used as an index of self-absorption for photons with different energies.

Therefore, one can write:

15 $$ R_{\text{P}} = \, f\left( {\mu_{\text{i}} } \right) \, \to \, f\left( {Z_{\text{R}} } \right) \, \to \, f\left( {\varepsilon_{\text{s}} } \right) $$

For the set of the secondary standards with the same specific activity of the ^210^Pb radionuclide, the total detection activity—*ε*_m_ can be calculated from Eq. (8), whereas the constant values for a given geometry—*ε*(*E*) can be obtained from the LabSOCS software, assuming no self-absorption of the material. On the basis of these calculations the self absorption coefficients—*ε*(*s*) can be calculated from Eq. (2).The results of such calculations are shown in the Table 2.

**Table 2:** Calibration of the system for ^210^Pb detection efficiencies

Standard reference material	*ε*_m_(*E*)	*ε*(*E*)	*ε*(*s*)	*R*_P1_ (911 keV/209 keV)	*R*_P2_ (609 keV/295 keV)
Soil Cu	0.14553	0.1848	0.7875	1.608	0.928
Soil Cu + 0.1 % Hg_2_Cl_2_	0.14462	0.1848	0.7826	1.644	0.943
Soil Cu + 0.2 % Hg_2_Cl_2_	0.14273	0.1848	0.7724	1.728	0.954
Soil Cu + 0.3 % Hg_2_Cl_2_	0.14077	0.1848	0.7618	1.766	0.973
Soil Cu + 0.4 % Hg_2_Cl_2_	0.14056	0.1848	0.7606	1.829	0.996
Soil Cu + 0.5 % Hg_2_Cl_2_	0.13976	0.1848	0.7563	1.895	1.006
Soil Cu + 0.6 % Hg_2_Cl_2_	0.13916	0.1848	0.7530	1.970	1.025
Soil 327	0.14828	0.1848	0.8024	1.660	0.909
Soil 327 + 0.05 % Pb	0.14734	0.1848	0.7973	1.703	0.940
Soil 327 + 0.10 % Pb	0.14661	0.1848	0.7933	1.758	0.986
Soil 327 + 0.15 % Pb	0.14507	0.1848	0.7850	1.811	1.010
Soil 327 + 0.20 % Pb	0.14424	0.1848	0.7805	1.887	1.054
Soil 327 + 0.30 % Pb	0.14342	0.1848	0.7761	1.968	1.065
Soil 327 + 0.50 % Pb	0.14209	0.1848	0.7689	1.997	1.095

The dependence of such calculated *ε*(*s*) values on the activity ratios—*R* for the chosen pairs of γ-lines is shown in Fig. 1.

As is evident for the examined soil samples in the wide range of their contamination with heavy metals, one can observe the linear relationship between the self absorption coefficient of the soft γ radiation of ^210^Pb and and Rp1 or Rp2 values. Therefore this relationship can be used as an easy self-absorption correction method without chemical analysis of the samples.

In order to check the usefulness of this method, we applied it to the determination of the ^210^Pb activity in other reference materials with certified values, or in samples with ^210^Pb activity determined on the basis of its daughter-^210^Po. The results are summarized in Table 3.

**Table 3:** Determination of ^210^Pb in different solid environmental samples with proposed self-absorption correction method

Sample	*R*_P1_	*ε*(*s*)_1_	*Ε*_m_(*E*)_1_	*A*_1_ (Bq/kg)	*R*_P2_	*ε*(*s*)_2_	*Ε*_m_(*E*)_2_	*A*_2_ (Bq/kg)	*A*_R_ (Bq/kg)	Relative deviation
IAEA-Phosphogypsum-434	1.9512	0.7764	0.1435	661.9	1.0474	0.7750	0.1432	663.2	680.0	0.026
IAEA-Sediment-368	1.9319	0.7781	0.1438	23.9	1.0683	0.7699	0.1423	24.2	23.2	−0.037
IAEA-Soil-375	1.7185	0.7966	0.1472	36.4	0.9918	0.7886	0.1457	36.7	36.2	−0.010
IAEA-Soil-6	1.7931	0.7901	0.1460	130.1	1.0048	0.7854	0.1451	130.9	137.6	0.052
SRM-Sediment-405	1.5527	0.8110	0.1499	52.5	0.9377	0.8018	0.1482	53.1	52.8	0.000
SRM-Su1-a	1.9919	0.7729	0.1428	20.6	1.0982	0.7626	0.1409	20.9	19.3	−0.079

As is evident from Table 3 the relative deviation of the results obtained by the proposed method does not exceed 8 % and it confirm its validity.

## Conclusion

In all solid environmental samples together with the very important ^210^Pb radionuclide there are other natural radionuclides. Some of them emit at least one of the pair of γ-photons with different energies. Simultaneous determination of the ratios of their γ-line activities can be a valuable method for searching for small chemical changes in the examined matrixes. We have proved this for at least following radionuclides: 228Ac emitting with sufficient efficiency photons with energies 209 and 911 keV, or a pair of 214Pb–214Bi with γ-ray energies of 252 and 609 keV can be used for simultaneous self-absorption correction in the determination of the another soft-γ emitter—^210^Pb.
